# Associations of ischemic heart disease with brain glymphatic MRI indices and risk of Alzheimer's disease

**DOI:** 10.1016/j.tjpad.2024.100045

**Published:** 2025-01-01

**Authors:** Ming-Liang Wang, Meng-Meng Yu, Zheng Sun, Jun-Jie Zhang, Jing-Kun Zhang, Xue Wu, Xiao-Er Wei, Yue-Hua Li

**Affiliations:** a Department of Radiology, Shanghai Sixth People's Hospital Affiliated to Shanghai Jiao Tong University School of Medicine, No. 600, Yi Shan Road, Shanghai 200233, China; b Department of Radiology, Zhongshan Hospital, Fudan University, Shanghai, China; c Cardiovascular Research Institute, University of California San Francisco, San Francisco, CA, USA; d Institute for Global Health Sciences, University of California San Francisco, San Francisco, CA, USA

**Keywords:** Ischemic heart disease, Alzheimer's disease, Cognitive impairment, MRI, Glymphatic

## Abstract

**Background:**

The impact of ischemic heart disease (IHD) on the brain glymphatic MRI indices and risk of Alzheimer's disease (AD) remains largely unclear. This study aimed to investigate the associations between IHD, brain glymphatic MRI indices and risk of AD.

**Methods:**

A total of 1385 non-dementia subjects (55.2 % male, mean age 73.53) were included. Diffusivity along the perivascular space (DTI-ALPS), free water (FW) and choroid plexus volume were used to reflect glymphatic function. The associations of IHD with MRI derived glymphatic indices, PET amyloid, tau and cognitive performance were explored by multiple regression analysis. IHD were tested as predictors of clinical progression using cox proportional hazards modeling. The mediation effect of MRI derived glymphatic indices on the relationship between IHD and cognitive changes was investigated.

**Results:**

Individuals with IHD exhibited glymphatic dysfunction revealed by lower DTI-ALPS (*p* = 0.035), higher FW (*p* < 0.001), and higher choroid plexus volume (*p* = 0.019). IHD had poorer cognitive performance in MMSE (*p* = 0.022), ADNI-MEM (*p* = 0.001) and ADNI-MF (*p* = 0.006), and more amyloid deposition (*p* = 0.007). IHD had a higher diagnostic conversion risk (HR = 1.321, 95 % CI = 1.003–1.741). IHD was associated with longitudinal cognitive decline in all cognitive tests (*p* < 0.05 for all) and FW (β = 0.012, 95 % CI 0.001, 0.023, *p* = 0.038). FW demonstrated an indirect effect (β = -0.0009, 95 % CI: -0.0034, -0.0001) and mediated 13.85 % effect for the relationship between IHD and ADNI-EF decline.

**Conclusion:**

IHD is independently associated with AD risk, and brain glymphatic dysfunction may partially mediate this relationship.

## 1 Introduction

Ischemic heart disease (IHD) has been demonstrated to be correlated with the poor brain health and development of dementia, especially vascular dementia [[1], [2], [3]]. The association between IHD and dementia may be attributed to shared risk factors, including advanced age, smoking, hypertension, elevated cholesterol levels, and diabetes mellitus [4,5]. Nevertheless, the relationships between IHD and Alzheimer's disease (AD) remained a topic of debate. While some studies have indicated a connection between IHD and AD [[6], [7], [8]], others have found no such association [9,10]. Despite various proposed pathways in the heart-brain connection [11], the exact causal mechanisms or common pathways by which IHD may cause AD remain to be elucidated.

The concept of brain glymphatic system was introduced by Iliff et al. in 2012 [12], suggesting that cerebrospinal fluid enters the brain parenchyma via arterial perivascular spaces, interacts with the brain interstitial fluid, and exits the brain parenchyma through venous perivascular spaces. This system is believed to be crucial for waste clearance in the brain and may have implications for neurodegenerative diseases [13]. Glymphatic failure may even serve as a final common pathway to dementia [14]. Studies on Alzheimer's disease have revealed impaired glymphatic function in mouse models of the disease [15]. The pulsation of the cerebral artery has been posited as a key factor in driving the brain glymphatic system [16]. As cerebral artery pulsation was prompted by the cardiac output, the abnormality of heart and the connecting artery may lead to dysfunction in the brain glymphatic system. Previous research has indicated a decrease in cerebrospinal fluid flow in individuals with hypertension, attributed to diminished artery pulsation [17]. At present, the potential impact of IHD on brain glymphatic function remains unexplored.

Recent advancements in neuroimaging have shown promise in assessing glymphatic function in humans through intrathecal or intravenous administration of gadolinium-based contrast agent by MRI [18,19]. However, the two approaches were deemed to be somewhat invasive. Promising noninvasive MRI-based methods included calculation of diffusion tensor image analysis along the perivascular space (DTI-ALPS) [20], assessment of the fractional volume of white matter free water (FW) from a bi-tensor DTI model [21], and measurement of choroid plexus volume [22]. These techniques have demonstrated efficacy in assessing the brain glymphatic system and have been applied in studies on AD [[23], [24], [25]]. To the best of our knowledge, no previous study has investigated the brain glymphatic function in IHD, and its potential role in the relationship between IHD and AD.

In this study, we hypothesize that (1) IHD is independently linked to an increased risk of AD and cognitive decline, (2) individuals with IHD exhibit impaired glymphatic system function as evidenced by DTI-ALPS, FW, and choroid plexus volume compared to those without IHD, and (3) brain glymphatic MRI indices play a mediating role in the association between IHD and cognitive impairment. This study aims to elucidate the connection between IHD and AD, as well as introduce a novel imaging biomarker and potential therapeutic target for reducing the risk of AD in patients with IHD.

## 2 Methods

### 2.1 Study sample

Data used in this study were obtained from the Alzheimer's Disease Neuroimaging Initiative (ADNI) database. The detailed information can be found at http://adni.loni.usc.edu. The ADNI study was approved by the institutional review boards of all participating institutions. Written informed consent was obtained from all the participants or their authorized representatives in accordance with the Declaration of Helsinki.

**Fig. 1:**
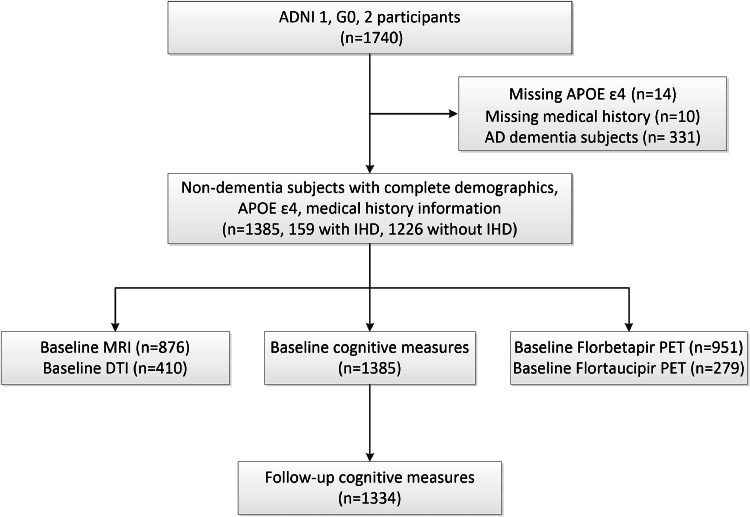
Flowchart of how the study samples were derived from the Alzheimer's Disease Neuroimaging Initiative. AD, Alzheimer disease; ADNI, Alzheimer's Disease Neuroimaging Initiative; APOE, apolipoprotein E; DTI, diffusion tensor imaging; IHD, ischemic heart disease; MRI, magnetic resonance imaging.

Fig. 1 illustrates the comprehensive sample selection process employed in the present study. A total of 1740 subjects were enrolled in the ADNI cohort (ADNI 1, G0, 2). Individuals lacking medical history, Apolipoprotein E (APOE) ɛ4 genotype, or AD dementia subjects were excluded. To sum up, this study included 1385 participants, comprising 159 individuals with IHD and 1226 without IHD. The initial data of MRI and PET were used for analysis. At follow-up, all available cognitive measures were collected for further analysis.

A history of IHD was determined in the screening assessment using ICD-10 codes, including angina pectoris, previous myocardial infarction, or any manifestation of IHD not resulting in infarction. Potential covariates included age, sex, education, right handedness, APOE ε4, hypertension, diabetes mellitus, hyperlipidemia, smoking, atrial fibrillation, and heart failure, as they were known to be related to both IHD and cognitive outcomes. History of hypertension, diabetes mellitus, hyperlipidemia, atrial fibrillation, and heart failure were obtained based on participants’ medical history. APOE genotype status was categorized as carriers or non-carriers of the ε4 allele. Data on age, sex, education, right handedness and smoking was collected through face-to-face interviews.

### 2.2 Assessment of cognitive function and progression

Mini-Mental State Examination (MMSE) was used to reflect global cognitive performance. Memory function and executive function were further investigated, as they were both reported in IHD [26]. ADNI memory composite score (ADNI-MEM) was used to asses memory function, which consisted of the Rey Auditory Verbal Learning task, word list learning and recognition tasks from ADAS-Cog, recall from Logical Memory I of the Wechsler Memory Test–Revised, and the 3-word recall item from the MMSE [27]. Executive function was evaluated by ADNI-EF, which was based on Category Fluency, Digit Span Backwards, Trail-Making Test Parts A and B, Wechsler Adult Intelligence Scale–Revised Digit–Symbol Substitution, and Clock Drawing items [28]. The baseline and all available follow-up cognitive test data were gathered for all included participants.

Participants were classified as cognitively normal (CN), mild cognitive impairment (MCI) or AD dementia at each visit. CN subjects had an MMSE score of 24–30 and a clinical dementia rating (CDR) score of 0. MCI subjects were diagnosed with an MMSE score of 24–30, a global CDR of 0.5, and a memory box score of at least 0.5. AD dementia subjects were diagnosed with an MMSE score of 20–26 and a global CDR≥0.5. Subjects diagnosed with early mild cognitive impairment or late mild cognitive impairment in the ADNI-2 study were all categorized as having MCI.

Progression of cognitive impairment at follow-up was defined as conversion from CN to MCI/AD dementia or from MCI to AD dementia. Participants who remained stable or reverted from MCI to CN were classified as showing no progression. The final diagnosis was determined based on the participant's status at their last visit for survival analysis. The time of progression was defined as the interval between the participant's visit date when conversion occurred and the baseline visit date. The time variable was encoded in months for further analysis.

### 2.3 Brain imaging and analysis

All subjects included in the analysis underwent MRI scanning using the ADNI 3T MRI scanning protocol from manufacturers GE, Philips, or Siemens. There was no statistical difference in the distribution of MRI manufacturers between the IHD and non-IHD groups (Table S1). White matter hyperintensity (WMH) volume, grey matter volume, white matter volume, cerebrospinal fluid volume and total intracranial volume were obtained with the methodology detailed on the ADNI website (http://adni.loni.usc.edu, “4-Tissue Segmentation Methods for ADNI MR Scans.pdf”). The volumes of the choroid plexus and hippocampus were extracted by FreeSurfer version 5.1 (http://adni.loni.usc.edu, “UCSF FreeSurfer Methods.pdf”). All the segmentation procedure passed the quality check and there were no abnormal values.

The DTI-ALPS, mean cerebral white matter FW, and Peak width of skeletonized mean diffusivity (PSMD) were derived from the DTI dataset. The DTI-ALPS was computed using the formula: DTI-ALPS = mean (Dx-proj, Dx-assoc)/mean (Dy-proj, Dz-assoc). The bilateral average of DTI-ALPS values was utilized for subsequent analyses. A higher DTI-ALPS ratio indicated increased water diffusivity within the perivascular spaces. The mean cerebral white matter free water metric was calculated using the script by the MarkVCID projects (https://markvcid.partners.org/markvcid1-protocols-resources). PSMD, which could reflect global white matter injury [29], was calculated in a fully automated fashion [30] (version 1.83; www.psmd-marker.com). Further details on the calculation methods were available in the supplementary material.

Brain amyloid and tau deposition were assessed by florbetapir (AV-45) PET and flortaucipir (AV-1451) PET. The detailed PET acquisition procedures could be obtained from the ADNI database (http://adni.loni.usc.edu, “PET Technical Procedures Manual: FDG (glucose metabolic imaging), Florbetapir or Florbetaben (Amyloid Imaging), AV-1451 (Tau Imaging)”). We used the processed results of UC Berkeley and Lawrence Berkeley National Laboratory. The mean florbetapir SUVR and meta temporal flortaucipir SUVR were collected. Further details were available in the supplementary material.

### 2.4 Statistical analysis

Normality was evaluated by the Shapiro–Wilk test and visual examination of histograms. Data were described as mean (standard deviation) for normally distributed variables, median with interquartile range for non-normally distributed variables, and number (percentage) for categorical variables. Baseline demographics, brain imaging markers and cognitive measures were compared between IHD group and non-IHD group. Quantitative variables were compared using Student's *t*-test or Mann-Whitney U test, while qualitative variables were compared using the chi-squared test.

The Kaplan–Meier method with log-rank tests was used to compare the incidence of clinical progression between IHD and non-IHD groups. Multivariable Cox proportional hazard regression model was conducted to estimate the hazard ratio (HR) of IHD in relation to clinical progression. The Cox proportional hazards assumption was assessed with the Schoenfeld residuals test. Individuals who did not develop MCI/AD dementia were censored at the time of their last evaluation. To investigate the joint effect of IHD with APOE ε4, diabetes mellitus, hypertension, or hyperlipidemia on clinical progression, we classified the participants into four groups according to joint exposures to IHD and APOE ε4, diabetes mellitus, hypertension, or hyperlipidemia, and repeated the multivariable Cox proportional models.

Additionally, multivariable linear regression analyses were employed to examine the associations between IHD, brain glymphatic MRI indices, PET measures and cognitive function. Assumptions of linearity, normality, independence, and variance homogeneity were met for the multivariable linear regression model.

To examine clinical progression in detail, linear mixed-effects (LME) models were utilized with MMSE, ADNI-MEM, and ADNI-EF as dependent variables and IHD, time, and the interaction term IHD × time as predictors. The LME models included random intercepts and slopes for time and an unstructured covariance matrix for the random effects. The primary focus was on the interaction term IHD × time, which assessed whether IHD moderated the relationship between time and cognitive performance decline. The slopes of MMSE, ADNI-MEM, and ADNI-EF were obtained. The regression model results are shown as beta coefficients, 95 % confidence intervals (CIs) and *p*-values. Furthermore, mediation analyses were conducted to examine the potential mediating role of brain glymphatic indices in the relationship between IHD and cognitive decline slope using the SPSS PROCESS module [31].

To test the robustness and potential variations across subgroups, we replicated the linear regression and LME analyses in CN/MCI groups, male/female groups and APOE ε4 (-) /APOE ε4 (+) groups. All statistical analyses were carried out with IBM SPSS Statistics for Windows, version 20.0 or R software (Version 4.3.0; Vienna, Austria). Statistical significance was determined using a two-tailed *p*-value threshold of <0.05.

## 3 Results

### 3.1 Baseline characteristics of study participants

Among the 1385 non-dementia subjects at baseline, the mean age was 73.53 years (SD 7.01) and 55.2 % were men. Table 1 shows the baseline characteristics of the study participants. 522 (37.7 %) subjects were cognitively normal and 863 (62.3 %) subjects were MCI. Compared with non-IHD subjects, IHD subjects were older (*p* < 0.001), less educated (*p* *=* 0.032) and more likely to be male (*p* < 0.001). IHD subjects had a higher prevalence of smoking history (*p* *=* 0.035), and comorbidities including hypertension, diabetes mellitus, hyperlipidemia, atrial fibrillation, and heart failure (*p* < 0.001 for all). There was no statistical difference in APOE ɛ4 (*p* *=* 0.309).

**Table 1:** Baseline characteristics in IHD and non-IHD group.

Characteristics	Total *n* = 1385	Non-IHD *n* = 1226	IHD *n* = 159	*P* value
Age (years)	73.53 (7.01)	73.19 (7.01)	76.13 (6.50)	<0.001*
Male sex, n ( %)	765 (55.2)	644 (52.5)	121 (76.1)	<0.001*
Education (years)	16.09 (2.79)	16.15 (2.81)	15.65 (2.62)	0.032*
Right handedness, n ( %)	1254 (90.5)	1112 (90.7)	142 (89.3)	0.572
APOE ε4 carrier, n (%)	584 (42.2)	511 (41.7)	73 (45.9)	0.309
Smoking, n (%)	547 (39.5)	472 (38.5)	75 (47.2)	0.035*
Hypertension, n (%)	686 (49.5)	581 (47.4)	105 (66.0)	<0.001*
Diabetes mellitus, n (%)	127 (9.2)	97 (7.9)	30 (18.9)	<0.001*
Hyperlipidemia, n (%)	630 (45.5)	517 (42.2)	113 (71.1)	<0.001*
Atrial fibrillation, n (%)	63 (4.5)	43 (3.5)	20 (12.6)	<0.001*
Heart failure, n (%)	13 (0.9)	5 (0.4)	8 (5.0)	<0.001*
**Baseline diagnosis**				0.738
CN, n (%)	522 (37.7)	464 (37.8)	58 (36.5)	
MCI, n (%)	863 (62.3)	762 (62.2)	101 (63.5)	

Values are shown as mean (SD) for the quantitative variables and as frequency (percentage) for the qualitative variables. Data of age, education were compared by *t*-test. Other variables were compared using the chi-squared test.* *p* < 0.05. Abbreviations: APOE, apolipoprotein E; CN, cognitively normal; IHD, ischemic heart disease; MCI, mild cognitive impairment.

### 3.2 Brain imaging markers and cognitive measures in IHD and non-IHD group

Table 2 presents the comparisons of brain imaging markers and cognitive measures between IHD and non-IHD group. Compared with non-IHD, IHD group had higher intracranial volume (*p* = 0.016), higher cerebrospinal fluid volume (*p* < 0.001), higher choroid plexus volume (*p* = 0.019), lower DTI-ALPS (*p* = 0.035), higher FW (*p* = 0.035) and higher brain amyloid SUVR (*p* = 0.007). There was no statistical difference in grey matter volume, white matter volume, hippocampus volume, WMH volume or PSMD (*p* > 0.05 for all). IHD group had poorer performance in MMSE (*p* = 0.022), ADNI-MEM (*p* = 0.001) and ADNI-EF (*p* = 0.006) than non-IHD group. During a median follow-up of 36 months, a total of 459 subjects (34.4 %) converted to MCI/AD dementia, including 71 IHD subjects (46.7 %) and 388 non-IHD subjects (32.8 %).

**Table 2:** Group comparisons of brain imaging markers and cognitive measures.

Characteristics	Total	Non-IHD	IHD	*P* value
**MRI measures**	*n* = 876	*n* = 801	*n* = 75	
Intracranial volume (cm^3^)	1199 (120)	1196 (121)	1231 (102)	0.016*
Grey matter volume (cm^3^)	587.7 (53)	587.0 (53)	595.7 (49)	0.176
White matter volume (cm^3^)	471.4 (60)	471.2 (61)	474.2 (54)	0.679
Cerebrospinal fluid volume (cm^3^)	333.0 (57)	330.7 (56)	357.6 (53)	<0.001*
WMH volume (cm^3^)	7.02 (10.15)	6.88 (10.19)	8.54 (9.58)	0.176
HP volume (cm^3^)	6.41 (0.89)	6.41 (0.89)	6.44 (0.91)	0.788
Choroid plexus volume, (cm^3^)^#^	4.16 (0.86)	4.14 (0.87)	4.40 (0.75)	0.019*
**DTI measures**	*n* = 410	*n* = 373	*n* = 37	
PSMD, 10^–4^mm^2^/s	3.00 (0.94)	3.00 (0.96)	3.04 (0.76)	0.827
DTI-ALPS	1.14 (0.16)	1.15 (0.16)	1.09 (0.13)	0.035*
Free Water	0.232 (0.037)	0.230 (0.034)	0.255 (0.044)	<0.001*
**AV 45 PET measure**	*n* = 951	*n* = 858	*n* = 93	
Florbetapir SUVR	1.18 (0.24)	1.17 (0.24)	1.24 (0.27)	0.007*
**AV 1451 PET measure**	*n* = 279	*n* = 255	*n* = 24	
Flortaucipir SUVR	1.30 (0.25)	1.30 (0.25)	1.33 (0.24)	0.517
**Cognition performance**	*n* = 1385	*n* = 1226	*n* = 159	
Baseline MMSE	28.14 (1.75)	28.18 (1.73)	27.84 (1.85)	0.022*
Baseline ADNI-MEM	0.55 (0.85)	0.57 (0.85)	0.34 (0.84)	0.001*
Baseline ADNI-EF	0.44 (0.87)	0.46 (0.88)	0.26 (0.83)	0.006*
**Clinical progression**	*n* = 1334	*n* = 1182	*n* = 152	
Follow-up, month	36 (24, 84)	42 (24, 84)	36 (18, 84)	0.370
Conversion to MCI/AD, n (%)	459 (34.4)	388 (32.8)	71 (46.7)	

Values are shown as mean (SD) or median (interquartile range) for the quantitative variables and as frequency (percentage) for the qualitative variables. Data of follow-up month was compared by Mann-Whitney *U* test. Other variables were compared using the *t*-test. * *p* < 0.05. ^#^The total available choroid plexus data were 841 (771 without IHD, 70 with IHD). Abbreviations: AD, Alzheimer disease; ADNI-EF, ADNI executive function score; ADNI-MEM, ADNI memory composite score; ALPS, along perivascular spaces; CN, cognitively normal; DTI, diffusion tensor imaging; HP, hippocampus; IHD, ischemic heart disease; MCI, mild cognitive impairment; MMSE, Mini-Mental State Examination; PSMD, peak width of skeletonized mean diffusivity; SUVR, standardized uptake value ratio, WMH, white matter hyperintensity.

### 3.3 IHD and risks of diagnostic conversion into MCI/AD dementia

**Fig. 2:**
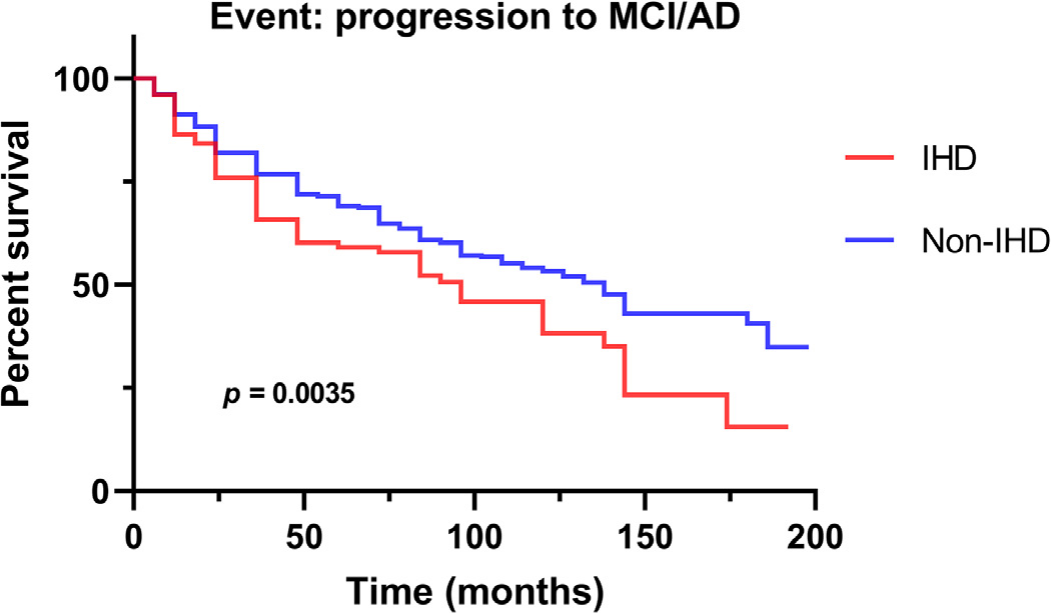
Kaplan‒Meier curve of diagnostic conversion. Individuals with IHD had a higher diagnostic conversion risk from CN to MCI/ AD dementia or from MCI to AD dementia than Non-IHD subjects (log-rank *p* = 0.0035). AD, Alzheimer disease; IHD, ischemic heart disease; MCI, mild cognitive impairment.

The Kaplan‒Meier curve of diagnostic conversion is shown in Fig. 2. Individuals with IHD exhibited a higher diagnostic conversion risk compared to individuals without IHD (log-rank *p* = 0.0035). Further Cox regression analyses revealed that IHD was associated with a higher risk of diagnostic conversion (HR = 1.321, 95 % CI = 1.003–1.741, *p* = 0.048), after adjusting for age, sex, education, right handedness, APOE ε4, hypertension, diabetes mellitus, hyperlipidemia, smoking, atrial fibrillation, and heart failure.

Compared to those with neither IHD nor APOE ε4, there was an elevated progression rate to MCI/AD dementia in subjects with either IHD (HR = 1.591, 95 % CI =1.064 –2.379, *p* = 0.024) or APOE ε4 (HR = 2.724, 95 % CI = 2.213–3.352, *p* < 0.001) alone; individuals with both IHD and APOE ε4 had tripled risk of MCI/AD (HR = 3.17, 95 % CI = 2.207–4.554, *p* < 0.001). Relative to those with neither IHD nor diabetes mellitus, there was an elevated progression rate to MCI/AD dementia in subjects with both IHD and diabetes mellitus (HR = 1.775, 95 % CI = 1.048–3.007, *p* = 0.033). Similarly, comorbid IHD and hypertension increased by 1.5-fold the risk of MCI/AD compared with those with neither IHD nor hypertension (HR = 1.532, 95 % CI = 1.088–2.159, *p* = 0.015) (Supplementary Fig. 1).

### 3.4 Association of IHD with brain imaging markers and cognitive decline

IHD was associated with FW [β = 0.013 (0.001, 0.024), *p* = 0.032], after controlling for age, sex, education, right handedness, APOE ε4, hypertension, diabetes mellitus, hyperlipidemia, smoking, atrial fibrillation, and heart failure. However, there was no statistical association between IHD and DTI-ALPS or choroid plexus volume (*p* > 0.05 for all) (Table 3).

**Table 3:** Associations of IHD with glymphatic MRI indices.

Glymphatic MRI indices	β (95 % CI)	*P* value
DTI-ALPS	−0.027 (−0.082, 0.029)	0.345
Free Water	0.013 (0.001, 0.024)	0.032*
Choriod plexus volume, (cm^3^)	−0.014 (−0.274 0.247)	0.918

Models were adjusted for age, sex, education, right handedness, APOE ɛ4, hypertension, diabetes, hyperlipemia, smoking, atrial fibrillation, heart failure, and intracranial volume. *: *p* < 0.0.5. Abbreviations: ALPS, along perivascular spaces; APOE, apolipoprotein E; DTI, diffusion tensor imaging; IHD, ischemic heart disease.

After adjusting for age, sex, education, right handedness, APOE ε4, hypertension, diabetes mellitus, hyperlipidemia, smoking, atrial fibrillation, and heart failure, the cross-sectional analysis revealed no statistical association between IHD and cognitive measures (*p* > 0.05 for all). IHD was associated with amyloid SUVR [β = 0.06 (0.011, 0.108), *p* = 0.016]. In the follow-up analysis, LME models indicated a connection between IHD and cognitive decline as measured by MMSE [β = −0.0097 (−0.013, −0.0063), *p* < 0.001], ADNI-MEM [β = −0.0014 (−0.0024, −0.0006), *p* = 0.002] and ADNI-EF [β = −0.0018 (−0.0027, −0.00084), *p* < 0.001] (Table 4).

**Table 4:** Cross-sectional and longitudinal multiple linear regression of IHD with cognitive performance.

	β (95 % CI)	*P* value
**Baseline**		
Amyloid SUVR	0.06 (0.011, 0.108)	0.016 *
Tau SUVR	0.049 (−0.065, 0.164)	0.395
MMSE	−0.100 (−0.392, 0.192)	0.502
ADNI-MEM	−0.050 (−0.186, 0.087)	0.475
ADNI-EF	−0.034 (−0.173, 0.105)	0.632
**LME model**		
MMSE	−0.0097 (−0.013, −0.0063)	<0.001 *
ADNI-MEM	−0.0014 (−0.0024, −0.0006)	0.002*
ADNI-EF	−0.0018 (−0.0027, −0.00084)	<0.001 *

Models were adjusted for age, sex, education, right handedness, APOE ɛ4, hypertension, diabetes, hyperlipemia, smoking, atrial fibrillation, and heart failure. In the LME model, IHD*time was the effect of interest, as it reflected whether IHD moderated the relationship between time and cognitive decline. *: *p* < 0.0.5. Abbreviations: ADNI-EF, ADNI executive function score; ADNI-MEM, ADNI memory composite score; IHD, ischemic heart disease; MMSE, Mini-Mental State Examination; LME, linear mixed-effects model.

For the association of IHD with longitudinal cognitive changes, sensitivity analysis confirmed the persistence of these associations in both CN and MCI subjects (Table S3), as well as in male subjects (Table S4) and individuals possessing the APOE ε4 allele (Table S5). However, the association was only observed between IHD with MMSE, not ADNI-MEM or ADNI-EF in female subjects and subjects lacking the APOE ε4 allele. For the association of IHD with baseline PET measures, sensitivity analysis revealed the persistence of these associations in male subjects (Table S4) and individuals lacking the APOE ε4 allele (Table S5)

### 3.5 Mediation effect of brain glymphatic indices on IHD and cognitive decline

**Fig. 3:**
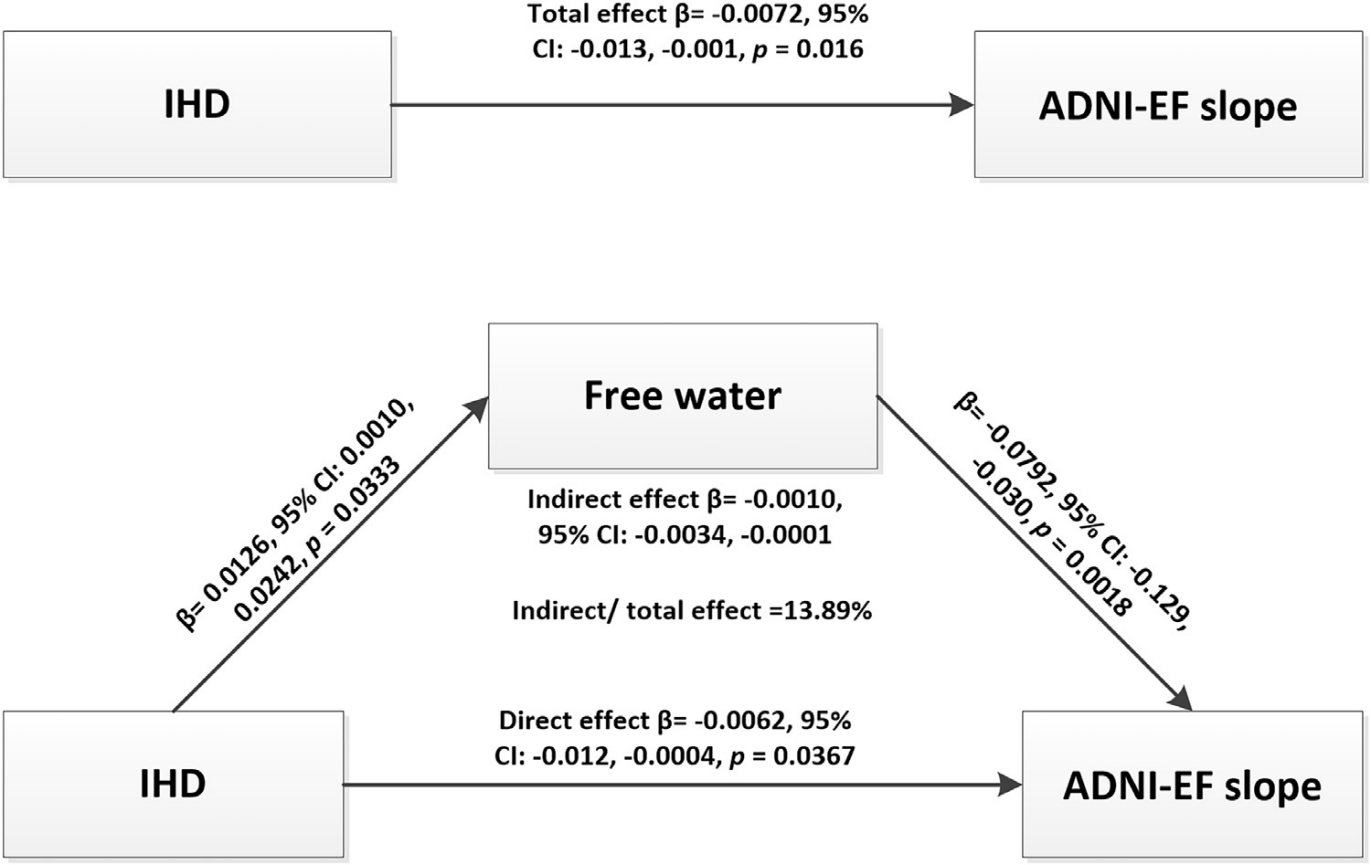
Mediation analysis. FW had a significant indirect effect (β = −0.0009, 95 % CI: −0.0034, −0.0001), mediating 13.85 % effect for the relationship between IHD and ADNI-EF slope. ADNI-EF, ADNI executive function score; IHD, ischemic heart disease.

Mediation analysis indicated that FW had a significant indirect effect (β = −0.0010, 95 % CI: −0.0034, −0.0001), accounting for 13.89 % of the relationship between IHD and ADNI-EF slope after adjusting for age, sex, education, right handedness, APOE ε4, hypertension, diabetes mellitus, hyperlipidemia, smoking, atrial fibrillation, and heart failure (Fig. 3). No mediation effect of FW was observed in the relationship between IHD and MMSE slope or ADNI-MEM slope.

## 4 Discussion

In this study, we investigated the influence of IHD on the brain glymphatic MRI indices and the risk of AD in non-dementia elderly. Our study indicated that IHD was significantly linked to an increased risk of AD and cognitive decline over a 36 months follow-up period. Additionally, IHD exhibited dysfunction in the brain glymphatic system revealed by MRI indices, with FW index playing a significant mediating role in the relationship between IHD and decline in ADNI-EF. These findings contribute to a proposed framework for understanding the role of the brain glymphatic system in the association between IHD and AD.

IHD and AD are two major health burdens among the elderly population globally [32], which share many risk factors. As mortality rates from IHD have decreased over the years, the prevalence of individuals with both chronic IHD and AD has increased [33]. Understanding the relationship between IHD and AD is crucial, as interventions for IHD in early adulthood may offer a new approach to preventing AD. Our findings align with previous research [[34], [35], [36]], showing a link between IHD and the risk of developing AD after accounting for common shared risk factors. Furthermore, combination with APOE ε4 (+), diabetes mellitus or hypertension increased the risk of MCI/AD in IHD. This was similar to a previous study using the same database [37]. As APOE ε4 was a well-established risk factor for AD progression, the combined effect was especially remarkable in subjects with APOE ε4 (+).

In the analysis of cognitive measures, our study demonstrated an association between IHD and cognitive decline in MMSE, ADNI-MEM and ADNI-EF tests. Previous studies have also indicated cognitive decline in MMSE, memory, and executive function [1,38,39]. In the sensitivity analysis, the associations persisted in both cognitively CN and MCI subjects, yet the statistical relationship was not observed in female subjects within the ADNI-MEM and ADNI-EF domains. It is hypothesized that this discrepancy may be attributed to the cognitive reserve present in female subjects, particularly in the realm of verbal memory [40]. Similarly, the statistical association was not evident in APOE ε4 (-) subjects within the ADNI-MEM and ADNI-EF domains, potentially due to the contradictory impact of APOE ε4 (-) on this association.

Furthermore, IHD was associated with baseline brain amyloid deposition after controlling for possible covariates including APOE ɛ4. A previous study has also revealed an association between coronary risk with cerebral amyloid deposition [41]. In the sensitivity analysis, the associations persisted in male subjects and individuals lacking the APOE ε4 allele, but not female subjects or individuals possessing APOE ε4 allele. Previous studies reported female had more tendency to brain amyloid than male [42,43]. We speculate the substantial impact of female gender and APOE ε4 (+) on brain amyloid deposition may overshadow the influence of IHD. The statistical associations were not evident in both cognitively CN and MCI subjects. This may be attributed to low statistical power resulting from a small number of IHD subjects in subgroup analysis. Future larger prospective study is needed to replicate our results.

Recent advancements in neuroimaging techniques have unveiled the existence of heart-brain connections [44,45], shedding light on the exploration of the correlation between IHD and AD. Our study found no significant variance in WMH volume between the IHD and non-IHD groups, which was similar to a previous research [46]. Additionally, there was no statistical difference in PSMD index, which may indicate a lack of global white matter damage in IHD. These findings suggest that cognitive decline and heightened AD susceptibility in individuals with IHD may not be directly linked to white matter damage. There was also no evidence of a statistically significant difference in hippocampal volume, grey matter volume or white matter volume in the IHD group compared to the non-IHD group. Other brain changes may be responsible for the observed association between IHD and increased risk of AD dementia.

After controlling for age, sex, education, APOE ɛ4, and intracranial volume, IHD was found to be correlated with white matter FW. FW represented water molecules that were not constrained or oriented, indicating the extracellular space [47]. The higher FW was thus proposed as a result of stagnation of fluid drainage due to impaired glymphatic function. Our study suggests that FW may be a sensitive indicator of glymphatic dysfunction in individuals with IHD. Previous research has also linked FW with cardiac atrial function [48] and cardiovascular biomarkers [49]. However, no such association was observed between IHD and DTI-ALPS or choroid plexus volume. The study of Kamagata K has proposed three indirect noninvasive MRI measures including perivascular space volume fraction, FW and DTI-ALPS could evaluate the different parts of brain glymphatic system shown in Fig. 1 in his study [23]. We speculated the glymphatic system was mainly impaired in the part revealed by FW in IHD.

The possible underlying mechanisms of brain glymphatic dysfunction in IHD are as follows. Individuals with IHD has been demonstrated to have reduced heart rate variability [50], and this may cause the decreased and irregular cardiac output to the brain during a heart attack [51,52]. Moreover, the panvascular medicine theory suggests that IHD often coexists with and negatively impacts cranial carotid arteriosclerosis [53,54]. All these factors may contribute to a gradual reduction in cerebral artery pulsation [55,56], ultimately may causing dysfunction of the brain glymphatic system. Left ventricular ejection fraction has been found to be correlated with AD-related cerebrospinal fluid biomarkers [57].

Moreover, our findings suggest that FW plays a crucial role as an indirect mediator in the relationship between IHD and ADNI-EF decline, but not MMSE decline or ADNI-MEM decline. This may be due to the fact that executive function is particularly vulnerable in IHD subjects [58]. Our study highlights the potential contribution of glymphatic dysfunction in the link between IHD and cognitive decline, indicating a promising avenue for the prevention of AD.

Our study is subject to several limitations. Firstly, the ADNI project utilized stringent inclusion and exclusion criteria, resulting in a sample that may not be representative of the general population. Consequently, caution is advised when generalizing the findings. Secondly, the diagnosis of IHD relied on self-reported medical history, introducing the potential for recall bias. Thirdly, longitudinal analysis of PET Aβ and tau changes was not performed due to significant missing longitudinal data. Thus, further research is recommended to address these gaps in knowledge. Fourthly, the limited number of subjects with available DTI data constrained the statistical power for additional analyses, and follow-up DTI data was not obtainable. A future study with a larger sample size is necessary to validate our findings. Additionally, the DTI data were sourced from three distinct manufacturers. Nonetheless, the acquisition protocol established by the ADNI project has been standardized, and there were no statistical differences in MRI manufacturer distribution between individuals with IHD and those without IHD. Previous research has also indicated minimal variability in DTI index calculations across various vendors [59].

## 5 Conclusions

In conclusion, our study indicates that IHD is independently linked to an increased risk of AD and cognitive decline. The presence of IHD is associated with impaired brain glymphatic function, as evidenced by glymphatic MRI indices. Furthermore, FW has a notable indirect impact, partially mediating the relationship between IHD and decline in ADNI-EF. These results provide evidence for the involvement of glymphatic dysfunction in the association between IHD and AD risk.

## ADNI consortia information

Data used in preparation of this article were obtained from the Alzheimer's Disease Neuroimaging Initiative (ADNI) database (adni.loni.usc.edu). As such, the investigators within the ADNI contributed to the design and implementation of ADNI and/or provided data but did not participate in analysis or writing of this report. A complete listing of ADNI investigators can be found at https://adni.loni.usc.edu/wp-content/uploads/how_to_apply/ADNI_Acknowledgement_List.pdf.

## Abbreviations

AD, Alzheimer disease; ADNI-EF, ADNI executive function score; ADNI-MEM, ADNI memory composite score; ALPS, along perivascular spaces; APOE, apolipoprotein E; CDR, clinical dementia rating; CN, cognitively normal; DTI, diffusion tensor imaging; HP, hippocampus; IHD, ischemic heart disease; LME, linear mixed-effects model; MCI, mild cognitive impairment; MMSE, Mini-Mental State Examination; PSMD, peak width of skeletonized mean diffusivity; SUVR, standardized uptake value ratio, WMH, white matter hyperintensity.

## Study funding

This study was further supported by 10.13039/501100012166National Key Research and Development Program of China (Grant No.2023YFF1204804, 2023YFF0722204), Shanghai Municipal Health Commission Research Project (Grant No. 20234Y0033), 10.13039/501100001809National Natural Science Foundation of China (Grant No. 82102033), 10.13039/501100013105Shanghai Rising-Star Program (Grant No. 24QA2708500), Shanghai Rising Stars of Medical Talent Youth Development Program [Grant No. SHWRS(2023)−062] and Shanghai Key Clinical Specialty (Grant No. shslczdzk03203). The funding sources had no role in study design, data collection, data analysis, data interpretation, or writing of the manuscript.

## CRediT authorship contribution statement

**Ming-Liang Wang:** Formal analysis, Funding acquisition, Investigation, Methodology, Project administration, Writing – original draft, Writing – review & editing. **Meng-Meng Yu:** Conceptualization, Formal analysis, Funding acquisition, Investigation, Methodology, Writing – review & editing. **Zheng Sun:** Formal analysis, Investigation, Methodology, Software, Writing – review & editing. **Jun-Jie Zhang:** Formal analysis, Investigation, Visualization, Writing – review & editing. **Jing-Kun Zhang:** Investigation, Methodology, Visualization, Writing – review & editing. **Xue Wu:** Investigation, Methodology, Visualization, Writing – review & editing. **Xiao-Er Wei:** Funding acquisition, Investigation, Project administration, Supervision, Writing – original draft, Writing – review & editing. **Yue-Hua Li:** Funding acquisition, Investigation, Methodology, Project administration, Supervision, Writing – original draft, Writing – review & editing.

## Declaration of competing interest

The authors declare that they have no known competing financial interests or personal relationships that could have appeared to influence the work reported in this paper.

## Appendix Supplementary materials

Image, application 1

Image, application 2
