# Supplementary material for: Associations of ischemic heart disease with brain glymphatic MRI indices and risk of Alzheimer's disease
Source: J Prev Alzheimers Dis. 2025 Jan 1;12(3):100045. doi: 10.1016/j.tjpad.2024.100045 (PMC12183944; doi:10.1016/j.tjpad.2024.100045)
Supplement: Supplementary file 1 [file mmc1.doc]

**Responses to the Reviewer’s Comments**

**Reviewer 1**

1. I thank the authors for providing their raw data for review, and this helps clarify the discrepancy. It might be worth the authors checking the ADNI table called ARM, which has a variable called ENROLLED. Negative values indicate that the individual failed screening and was thus not enrolled in the study. When I cross-reference the subjects in where the APOE data is missing from the raw data provided by the author with subjects marked as screen failure in ENROLLED by ADNI, there are a lot of matches. Thus it is likely that most of the potential participants missing APOE data were never actually enrolled in ADNI. I would suggest reviewing this data and updating the Methods text and Figure 1 flowchart to reflect this.

**Response: Thanks a lot for this important comment. After checking the “ARM” table, we found there were indeed a lot of subjects with demographic information but were not enrolled in the ADNI cohort. We also carefully reviewed the most recent “ADNIMERGE” table. The total enrolled subjects (ADNI 1, G0, 2) were 1740 and there were only 14 subjects without APOE data, which were correctly consistent with your findings. Then, 10 subjects lacking medical history information and 331 AD subjects were further excluded in this study. The total study subjects were still the original 1385 subjects, and the final results were not affected. We have updated the method text marked in yellow and revised the Figure 1 flowchart. Thanks again for your valuable suggestion.**

1. In the methods section, please indicate that your time variable is encoded in months, so that people can better interpret the coefficients in your model that are related to time.

**Response: We are thankful for this constructive comment. According to your suggestion, we have added the description “Time variable was encoded in months for further analysis” in the method part marked in yellow. Moreover, we also changed the expression of followup time in “years” into “months” , which could be more understandable. Thanks again for your kind comment.**

1. The authors state in the discussion "As the three markers reflect different aspects of the glymphatic system, we speculated the glymphatic system was mainly impaired in the part revealed by FW in IHD". From this sentence, it wasn't quite clear what the different aspects were that each image measure is assessing. I then looked at the authors reference 23, which was very helpful on this topic, in particular Figure 1. I would suggest the authors specifically point out this reference this figure in this part of the discussion to help readers understand the different aspects of glymphatic dysfunction that each imaging measure is potentially assessing.

**Response: We are thankful for this valuable comment. Indeed, the study of Kamagata K has proposed three indirect noninvasive MRI measures including perivascular space volume fraction, FW and DTI-ALPS could evaluate the different parts of brain glymphatic system shown in Figure 1 (reference 23). We speculated the glymphatic system was mainly impaired in the part revealed by FW in IHD. According to your suggestion, we have added this important information, and specially cited reference 23 Figure 1 in the “Discussion” part marked in yellow. Thanks again for your helpful comment.**

1. Table 3 - the p value for choroid plexus volume is missing a decimal point.

**Response: We are sorry for this clerical error. We have thoroughly checked our manuscript and corrected this clerical error marked in yellow. Thanks a lot for your valuable comments.**

**In conclusion, according to the comments, above is what we have modified in this manuscript. We appreciate for the Editor/Reviewer’s constructive comments earnestly and hope that the correction will meet with approval. Once again, thank you so much for your comments and suggestions.**
