# Supplementary material for: Associations of ischemic heart disease with brain glymphatic MRI indices and risk of Alzheimer's disease
Source: J Prev Alzheimers Dis. 2025 Jan 1;12(3):100045. doi: 10.1016/j.tjpad.2024.100045 (PMC12183944; doi:10.1016/j.tjpad.2024.100045)
Supplement: Supplementary file 2 [file mmc2.docx]

**Supplementary Material**

**eMethod 1.** Alzheimer’s Disease Neuroimaging Initiative (ADNI)

**eMethod 2.** The procedure of PSMD, DTI-ALPS and FW

**eMethod3.** The processing procedure of florbetapir (AV-45) PET and flortaucipir (AV-1451) PET

**Table S1.** The distribution of MRI manufacture in IHD and non-IHD groups

**Table S2.** DTI acquisition representative parameters in different MRI manufactures

**Table S3.** Linear regression analysis of IHD with cognitive performance in CN and MCI subjects

**Table S4.** Linear regression analysis of IHD with cognitive performance in male and female subjects

**Table S5.** Linear regression analysis of IHD with cognitive performance in APOE ε4 (-) and APOE ε4 (+) subjects

**Supplementary Fig.1** Figure legend

**eMethod 1.**

Data used in this study were obtained from the Alzheimer's Disease Neuroimaging Initiative (ADNI) database, led by Principal Investigator Michael W. Weiner, M (http://adni.loni.usc.edu). The ADNI project was launched in 2003 and was designed to measure the progression of mild cognitive impairment and early Alzheimer's disease by investigating serial MRI, PET, biological markers, and clinical and neuropsychological assessments. The ADNI study was approved by the institutional review boards of all participating institutions. Written informed consent was obtained from all the participants or their authorized representatives in accordance with the Declaration of Helsinki. The specific enrolment procedure and inclusion criteria for the different diagnostic categories in the ADNI cohort have been described previously [1].

**eMethod 2.**

Peak width of skeletonized mean diffusivity (PSMD) was used to reflect global white matter damage. In this regard, PSMD was calculated in a fully automated fashion (version 1.83; www.psmd-marker.com). First, FA images, nonlinearly registered with FNIRT (FMRIB Nonlinear Image Registration Tool) into standard space using the FMRIB FA template (1 mm), were projected onto the white matter (WM) skeleton, which was derived from the standard space template with a threshold FA value of 0.2. Then, the mean diffusion (MD) images were projected onto the skeleton using the FA-derived projection parameters. Finally, PSMD was calculated as the difference between the 95th and 5th percentiles of the MD voxel values within the WM skeleton [2].

The procedure for DTI-ALPS measurement has been well established. Firstly, the DTI parameters including fractional anisotropy (FA) map, and the diffusivity in the directions of x-axis (Dx), y-axis (Dy), and z-axis (Dz) were calculated by FMRIB Software Library (FSL) toolbox (version 5.0.1, https://fsl.fmrib.ox.ac.uk/fsl/fslwiki). Secondly, for each subject, the FA map was registered to the FA template (https://neurovault.org/images/1406/) using Advanced Normalization Tools (ANTs, https://stnava. github.io/ANTs/). The acquired transformation matrix was also applied to individual (Dxx, Dyy, Dzz) maps. Thirdly, four ROIs with a diameter of 5 mm were drawn at the areas of the projection neural fiber and association neural fiber at the level of the lateral ventricle bodies in the left and right hemispheres on the FA template. Then, the four ROIs were transferred to the individual FA template. The position of the resulting ROIs in the FA template space was visually checked for each participant. Finally, difusivities along the x-axis (Dxx), y-axis (Dyy), and z-axis (Dzz) were extracted for each ROI. The DTI-ALPS was calculated according to the following equation: DTI-ALPS = mean (Dx-proj, Dx-assoc)/mean (Dy-proj, Dz-assoc). The average value of the bilateral DTI-ALPS was used in further analyses. A higher ratio represented more water diffusivity along the perivascular spaces.

Maps of the fractional volume of free water (FW) were constructed from the DW images using a regularized bitensor model with the open-source script provided by the MarkVCID projects (https://markvcid.partners.org/markvcid1-protocols-resources). Briefly, the script contains the following steps: 1) the tissue compartment is modeled by a diffusion tensor characterizing the “tissue” molecules, as well as the fractional volume of the free-water compartment in each voxel, resulting in the FW fraction map, 2) the individual FA map obtained from dtifit is linearly and non-linearly registered to the standard FSL FA template space (FMRIB 1-mm FA template) using linear and nonlinear transformations, 3) the resulting transformation parameters are applied to the FW map, 4) a white matter (WM) mask is defined by thresholding the FSL FA template at a value of 0.3 to reduce cerebrospinal fluid partial volume contamination17, 5) an overall measure of mean FW is computed by superimposing the WM mask onto the individual coregistered FW fraction map and averaging values within these WM voxels. The fractional volume of the free water compartment (ie, the free water measure) reflects the relative contribution of free water in each voxel, ranging from 0 to 1.

**eMethod 3.**

The detailed florbetapir (AV-45) PET and flortaucipir (AV-1451) PET acquisition procedures could be obtained from the ADNI database (http://adni.loni.usc.edu, “PET Technical Procedures Manual: FDG (glucose metabolic imaging), Florbetapir or Florbetaben (Amyloid Imaging), AV-1451 (Tau Imaging)”). For the processing of florbetapir images, the florbetapir images were coregistered to the corresponding MRI and calculate the mean florbetapir uptake within the cortical regions including frontal, anterior/posterior cingulate, lateral parietal, and lateral temporal with the whole cerebellum as reference region [3]. For the processing of flortaucipir images, the flortaucipir images were coregistered to the corresponding MRI and calculate the mean florbetapir uptake within a tau metaROI including entorhinal, amygdala, parahippocampal, fusiform, inferior temporal and middle-temporal normalized to cerebellar-crus [4].

**References**

1. Petersen RC, Aisen PS, Beckett LA, Donohue MC, Gamst AC, Harvey DJ, et al. Alzheimer's Disease Neuroimaging Initiative (ADNI): clinical characterization. Neurology. 2010;74:201-9.
2. Baykara E, Gesierich B, Adam R, Tuladhar AM, Biesbroek JM, Koek HL, et al. A Novel Imaging Marker for Small Vessel Disease Based on Skeletonization of White Matter Tracts and Diffusion Histograms. Ann Neurol. 2016;80:581-92.
3. Landau SM, Lu M, Joshi AD, Pontecorvo M, Mintun MA, et al. Comparing positron emission tomography imaging and cerebrospinal fluid measurements of β-amyloid. Ann Neurol. 2013;74:826-36.
4. Jack CR, Jr., Wiste HJ, Weigand SD, et al. Age-specific and sex-specific prevalence of cerebral β-amyloidosis, tauopathy, and neurodegeneration in cognitively unimpaired individuals aged 50-95 years: a cross-sectional study. Lancet Neurol. 2017;16:435-44.

**Table S1 The distribution of MRI manufacture in IHD and non-IHD groups**

| **Diagnosis** | **MRI manufacture** | **Frequency** | ***P* value** |
| --- | --- | --- | --- |
| IHD | Siemens | 161 (42.3%) | 0.717 |
|  | General Electric | 181(48.5%) |  |
|  | Philips | 31 (8.3%) |  |
| Non-IHD | Siemens | 18 (48.6%) |  |
|  | General Electric | 17 (45.9%) |  |
|  | Philips | 2 (5.4%) |  |

Values are shown as frequency (percentage), and compared using the chi-square test. * *p* < 0.05. Abbreviations: IHD, ischemic heart disease.

**Table S2 DTI acquisition representative parameters in different MRI manufacture**

| Manufacturer | TR  (ms) | TE  (ms) | Flip angle | Slice thickness（mm） | Gradient direction | Diffusion sensitivity coefficient |
| --- | --- | --- | --- | --- | --- | --- |
| Siemens | 9600 | 56 | 90° | 2 | 54 | 1000 |
| GE | 9050 | 61.9 | 90° | 2 | 41 | 1000 |
| Philips | 9916 | 85.7 | 90° | 2 | 36 | 1000 |

Abbreviations: TR, repetition time; TE, echo time;

**Table S3 Cross-sectional and longitudinal multiple linear regression of IHD with cognitive performance in CN and MCI subjects**

|  | **CN** | | **MCI** | |
| --- | --- | --- | --- | --- |
|  | **β (95% CI)** | ***P* value** | **β (95% CI)** | ***P* value** |
| **Baseline** |  |  |  |  |
| Amyloid SUVR | 0.06 (-0.007, 0.127) | 0.008 | 0.055 (-0.012, 0.122) | 0.105 |
| Tau SUVR | -0.010 (-0.111, 0.092) | 0.851 | 0.063 (-0.159, 0.285) | 0.577 |
| MMSE | 0.082 (-0.230, 0.393) | 0.607 | -0.201 (-0.590, 0.188) | 0.310 |
| ADNI-MEM | 0.016 (-0.158, 0.189) | 0.861 | -0.110 (-0.263, 0.044) | 0.161 |
| ADNI-EF | -0.073 (-0.271, 0.126) | 0.472 | -0.017 (-0.190, 0.157) | 0.851 |
| **LME model** |  |  |  |  |
| MMSE | -0.0049 (-0.0084, -0.0014) | 0.006 * | -0.013 (-0.018, -0.0075) | <0.001* |
| ADNI-MEM | -0.0013 (-0.0025, -0.00002) | 0.046 * | -0.0015 (-0.0027, -0.00002) | 0.020 * |
| ADNI-EF | -0.0014 (-0.0027, -0.00005) | 0.043 * | -0.0021 (-0.0033, -0.0008) | 0.001* |

Models were adjusted for age, sex, education, right handedness, APOɛ4, hypertension, diabetes, hyperlipemia, smoking, atrial fibrillation, and heart failure. In the LME model, IHD*time was the effect of interest, as it reflected whether IHD moderated the relationship between time and cognitive decline. *: *p* < 0.0.5. Abbreviations: ADNI-EF, ADNI executive function score; ADNI-MEM, ADNI memory composite score; CN, Control; IHD, ischemic heart disease; MCI, mild cognitive impairment; MMSE, Mini-Mental State Examination; LME, linear mixed-effects model.

**Table S4 Cross-sectional and longitudinal multiple linear regression of IHD with cognitive performance in male and female subjects**

|  | **Male** | | **Female** | |
| --- | --- | --- | --- | --- |
|  | **β (95% CI)** | ***P* value** | **β (95% CI)** | ***P* value** |
| **Baseline** |  |  |  |  |
| Amyloid SUVR | 0.081 (0.022, 0.141) | 0.008 * | 0.003 (-0.084, 0.090) | 0.946 |
| Tau SUVR | 0.105 (-0.022, 0.231) | 0.104 | -0.042 (-0.277, 0.192) | 0.722 |
| MMSE | -0.047 (-0.393, 0.299) | 0.791 | -0.243 (-0.806, 0.321) | 0.398 |
| ADNI-MEM | -0.007 (-0.158, 0.144) | 0.925 | -0.199 (-0.483, 0.085) | 0.169 |
| ADNI-EF | -0.002 (-0.167, 0.164) | 0.982 | -0.137 (-0.406, 0.131) | 0.316 |
| **LME model** |  |  |  |  |
| MMSE | -0.0095 (-0.0137, -0.0054) | <0.001 * | -0.0095 (-0.0158, -0.0033) | 0.003 * |
| ADNI-MEM | -0.0022 (-0.0032, -0.0012) | <0.001 * | 0.00013 (-0.0016, 0.0019) | 0.888 |
| ADNI-EF | -0.0026 (-0.0036, -0.0015) | <0.001 * | -0.00004 (-0.0018, 0.0017) | 0.961 |

Models were adjusted for age, sex, education, right handedness, APOɛ4, hypertension, diabetes, hyperlipemia, smoking, atrial fibrillation, and heart failure. In the LME model, IHD*time was the effect of interest, as it reflected whether IHD moderated the relationship between time and cognitive decline. *: *p* < 0.0.5. Abbreviations: ADNI-EF, ADNI executive function score; ADNI-MEM, ADNI memory composite score; CN, Control; IHD, ischemic heart disease; MCI, mild cognitive impairment; MMSE, Mini-Mental State Examination; LME, linear mixed-effects model.

**Table S5 Cross-sectional and longitudinal multiple linear regression of IHD with cognitive performance in APOE ε4 (-) and APOE ε4 (+) subjects**

|  | **APOE ε4 (-)** | | **APOE ε4 (+)** | |
| --- | --- | --- | --- | --- |
|  | **β (95% CI)** | ***P* value** | **β (95% CI)** | ***P* value** |
| **Baseline** |  |  |  |  |
| Amyloid SUVR | 0.064 (0.005, 0.122) | 0.033 * | 0.057 (-0.028, 0.142) | 0.185 |
| Tau SUVR | 0.038 (-0.086, 0.162) | 0.547 | 0117 (-0.131, 0.366) | 0.351 |
| MMSE | -0.020 (-0.388, 0.349) | 0.917 | -0.204 (-0.680, 0.273) | 0.401 |
| ADNI-MEM | -0.002 (-0.176, 0.173) | 0.986 | -0.110 (-0.329, 0.109) | 0.324 |
| ADNI-EF | -0.104 (-0.293, 0.085) | 0.281 | 0.072 (-0.135, 0.278) | 0.497 |
| **LME model** |  |  |  |  |
| MMSE | -0.0079 (-0.0117, -0.0040) | <0.001 * | -0.011 (-0.017, -0.0045) | 0.001 * |
| ADNI-MEM | -0.00065 (-0.0017, 0.00043) | 0.241 | -0.0023 (-0.0038, -0.00082) | 0.002 * |
| ADNI-EF | -0.00068 (-0.0018, 0.00042) | 0.225 | -0.0037 (-0.0053, -0.0021) | <0.001 * |

Models were adjusted for age, sex, education, right handedness, APOɛ4, hypertension, diabetes, hyperlipemia, smoking, atrial fibrillation, and heart failure. In the LME model, IHD*time was the effect of interest, as it reflected whether IHD moderated the relationship between time and cognitive decline. *: *p* < 0.0.5. Abbreviations: APOE, apolipoprotein E; ADNI-EF, ADNI executive function score; ADNI-MEM, ADNI memory composite score; CN, Control; IHD, ischemic heart disease; MCI, mild cognitive impairment; MMSE, Mini-Mental State Examination; LME, linear mixed-effects model.

**
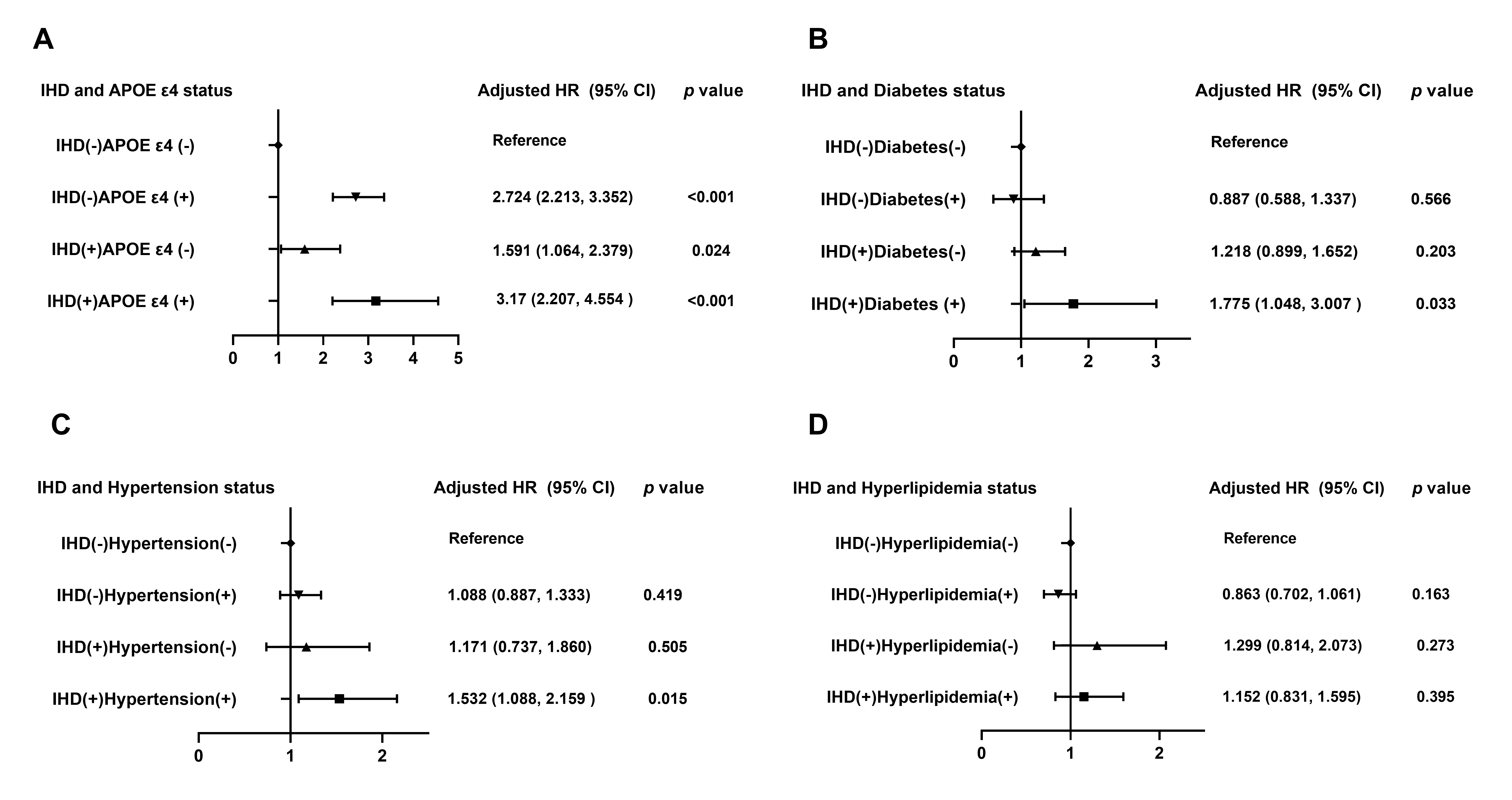
Supplementary Figure1 legend**

Joint effect of IHD with comorbidities and APOE ε4 allele on the risk of clinical progression from from CN to MCI/ AD dementia or from MCI to AD dementia. Forest plots show the hazard ratios (HRs) and 95% confidence intervals (Cls) derived from multi-variable Cox regression models grouped by different IHD and APOE ε4 status (A), different IHD and diabetes mellitus status (B), different IHD and hypertension status (C), different IHD and hyperlipidemia status (D). AD, Alzheimer disease; IHD, ischemic heart disease; MCI, mild cognitive impairment.
